# Functions of CsGPA1 on the hypocotyl elongation and root growth of cucumbers

**DOI:** 10.1038/s41598-018-33782-4

**Published:** 2018-10-22

**Authors:** Yan Yan, Wenna Zhang, Yansu Li, Chaoxing He, Lihong Gao, Xianchang Yu

**Affiliations:** 1 Beijing Key Laboratory of Growth and Developmental Regulation for Protected Vegetable Crops, China Agricultural University, 2 Yuanmingyuan West Road, Haidian District, Beijing, 100193 China; 2 The Institute of Vegetables and Flowers, Chinese Academy of Agricultural Sciences, 12 Zhongguancun South St, Haidian District, Beijing, 100081 China

## Abstract

G proteins regulate shoot, root, and epidermis development, as well as biotic stress tolerance in plants; however, most studies have examined model plants and less attention has been paid to the function of G proteins in horticultural plants. Here, we identified a G protein, *CsGPA1*, from cucumber and studied its function in seedling development of cucumbers. CsGPA1 is a peptide of 392 amino acids with a predicted molecular mass of 44.6 kDa. Spatiotemporal expression analysis found that endogenous *CsGPA1* was highly expressed in roots and young leaves. Immunohistochemical assay revealed that functional CsGPA1 was present in the plasma membranes of the epidermis and cortex, and in the cytosol of the endodermis, parenchyma, and vasculature of root meristematic cells. In comparison with wild-type seedlings, *CsGPA1*-overexpressing transgenic lines exhibited enhanced seed germination and earlier seedling development including hypocotyl elongation and root growth. In contrast, RNAi silencing of the *CsGPA1* gene inhibited seedling growth and development. Further study showed that CsGPA1 controled hypocotyl elongation and root growth via promoting cell size and the meristem of hypocotyl and root tip cells of cucumber plants. Our study expands the roles of G proteins in plants and helps to elucidate the mechanism by which cucumber regulates early seedling development.

## Introduction

Heterotrimeric G proteins form a family of GTP-binding proteins and function as signaling molecules that are activated after binding to GTP^1^. These proteins are localized to the plasma membrane, where they interact with downstream effectors^2^. G proteins are involved in the transduction of extracellular signals into intracellular responses. In the inactive state, a G protein typically exists as a trimer consisting of an α-subunit (Gα) bound to GDP, a β-subunit (Gβ), and a γ-subunit (Gγ). When a ligand binds to a G protein-coupled receptor, a conformational change occurs in the G protein, resulting in the exchange of the GDP for a GTP and the dissociation of Gα-GTP from the Gβγ dimer. The G-protein subunits remain active until the intrinsic GTPase activity of Gα results in the hydrolysis of GTP to GDP and the reassociation of the inactive trimer. The *Arabidopsis thaliana* genome contains canonical Gα and Gβ genes, *GPA1* and *AGB1*, and two genes that encode Gβγ, *AGG1* and *AGG2*^1^.

The Gα subunit is an important component of the heterotrimeric G-protein complex and is associated with the Ca^2+^/CaM signature, regulation of monomeric GTPases, phospholipase C- and D-mediated signaling, and the relay into secondary signaling pathways, which in turn lead to changes in the activity of specific proteins^3,4^_._ It plays important roles in the diverse process of plant growth and development. GPA1 in *Arabidopsis* is involved in seed germination and hypocotyl length^5–8^. *gpa1* mutants display the phenotype of fewer lateral roots and a shorter hypocotyl and blades^9,10^. In rice, Gα is required for normal shoot elongation and seed growth^11^. The *d1* mutant of rice exhibits dwarfism and produces erect leaves and small seeds^12^. Accumulating evidence indicates that GPA1 likely influences leaf, hypocotyl, and root growth by modulating cell division and proliferation. In addition, *GPA1* is also involved in the response to drought, heat, and cold abiotic stresses by transmitting the signal through downstream effectors including ion channels, phospholipases, kinases/phosphatases, and other GTPases. The interaction between GPA1 and other signaling pathways such as S1P (sphingosine-1-phosphate)^13^ and the ABA signaling pathway has been recently uncovered.

Cucumber (*Cucumis sativus* L.) is one of the main vegetables cultivated in China. There are several stress conditions that significantly affect cucumber production, such as cold stress during the winter in the northern regions. Cucurbitaceae is one of only a few eudicot families with internal as well as external phloem, and thus cucumber requires individual study to maximize agricultural success. Although the Gα subunit has been the subject of study in model plants, the cucumber Gα subunit has not previously been characterized. In this study, we identified a G-protein from cucumber and investigated its function in seedling growth and development.

## Materials and Methods

### Plant materials and growth conditions

Cucumber (*Cucumis sativus* cv. Xintai Mici) seeds were immersed in hot water (55 °C) for 4 h. The seeds were then placed in 9-cm diameter Petri dishes containing two layers of filter paper moistened with 5 mL sterilized distilled water. Plates were incubated for 6 d at 30 °C in darkness. Cucumber seedlings with two true leaves were transferred to a growth chamber and grown under a 14-h light (28 °C)/10-h dark (18 °C) photoperiod (600 mmol m^−2^ s^−1^ photosynthetic photon flux density) with 70% relative humidity.

### RNA extraction and cDNA synthesis

Total RNA was extracted from cucumber leaves using a total RNA isolation kit (Beijing Huayueyang, Beijing, China). The extracted RNA was treated with DNase A to remove residual DNA. First-strand cDNA was synthesized using a PrimeScript™ RT reagent Kit (TaKaRa Biotechnology, Dalian, China) with 2 µg RNA as the template. Quantitative real-time (qRT)-PCR was carried out using 96-well blocks in a 7500 Real Time PCR System (Applied Biosystems, Foster City, CA, USA). The 20-µL reaction volumes were prepared using SYBR® Green PCR Master Mix (Applied Biosystems) and appropriate primers (Supplementary Table 1). qRT-PCR was completed with 40 cycles of 95 °C for 5 s and 60 °C for 34 s. A melting curve analysis was used to verify the amplified product. The cycle threshold (Ct) values were converted to relative copy numbers using the ∆∆Ct method. Gene expression data were normalized against the expression of *CsActin*.

### Gene cloning

Primers were designed to amplify the *CsGPA1* gene using the published *CsGPA1* consensus sequence and the Cucumber Genome Database (http://www.icugi.org/cgi-bin/ICuGI/index.cgi). A 1,179-bp fragment was amplified by a polymerase chain reaction (PCR) using PrimeSTAR Max DNA Polymerase (TaKaRa) with the prepared cDNA as the template. The PCR program was as follows: 95 °C for 3 min and 35 cycles of 95 °C for 30 s, 52 °C annealing for 45 s, and 72 °C for 2 min.

To generate the over-expression construct, the *CsGPA1* coding region was PCR-amplified and cloned into the *Sma*I/*Xba*I sites of the pBI121 binary vector under the control of the 35 S promoter. To generate the RNA interference (RNAi) construct, a 129-bp sequence of *CsGPA1* cDNA was PCR-amplified and cloned into the pFGC1008 binary vector. To investigate the subcellular localization of CsGPA1, the encoding region of *CsGPA1* was PCR-amplified and inserted into the pUC vector (http://deepgreen.stanford.edu), through which a fusion protein comprising CsGPA1 and the green fluorescent protein (GFP) was generated. The sequences of the primers used to generate these constructs are listed in Supplementary Table 1.

### Cucumber transformation

Cucumber (cv. Xintai Mici) plants were transformed with *Agrobacterium tumefaciens*, as previously described^14^. Briefly, 2-d-old cotyledons were cut into two pieces. After removing the germs, the cotyledons were incubated in MS liquid medium containing *A*. *tumefaciens* LBA4404 cells harboring pBI121-antisense-*CsGPA1* and 35S::CsGPA1-GFP for 12 min at 28 °C. They were then placed on polarization medium (MS supplemented with 0.5 mg/L 6-BA and 1 mg/L ABA, pH 5.7–5.8). After culturing for 2 d at 28 °C in darkness, the cotyledons were transferred to polarization medium supplemented with 100 mg/L kanamycin and 500 mg/L carbenicillin. After 2–3 weeks, the shoots that differentiated from the cotyledons were transferred to rooting medium (MS supplemented with 100 mg/L kanamycin and 200 mg/L carbenicillin) to stimulate root growth. After 20–30 d, when the new roots were growing well in the rooting medium, the seedlings were exposed to weak light for 3–4 d and then transferred to pots containing vermiculite. The transgenic plants were cultivated in a phytotron under a 12-h light (25 °C)/12-h dark (18 °C) photoperiod (600 umol m^−2^s^−1^).

### Phylogenetics

CLUSTAL W and MEGA7 software was used to analyze the phylogenetic relationships between the *GPA1* genes from *C*. *sativus*, *Zea mays*, *Oryza sativa* subsp. *indica* (rice), *O*. *sativa* subsp. *japonica*, *Pisum sativum* (pea), *Solanum lycopersicum* (tomato), *Solanum tuberosum* (potato), *Glycine max* (soybean), *Spinacia oleracea* (spinach), *Sasa veitchii*, *Chusquea spectabilis*, *Phyllostachys bambusoides* (timber bamboo), *Picea abies* (Norway spruce), and *Arabidopsis thaliana* based on the encoded amino acid sequences (Thompson, Higgins & Gibson 1994). The phylogenetic tree was constructed using the neighbor-joining method of the MEGA7 software^15^.

### Seed germination rate

To determine the germination rate of the seeds from different transgenic cucumber plants, the second and third fruits were collected 40 d after pollination and ripened for 5 d. Seeds were collected and 20–30 were placed in 9-cm diameter Petri dishes containing two layers of filter paper moistened with 5 mL sterilized distilled water. Plates were incubated for 2 d at 30 °C in darkness. Seeds were considered germinated if the emerging radicle grew to 3 mm. At least three replicates, with 30 plants per replicate, were used for the germination rate analysis.

### Morphometry

The leaf blade width and hypocotyl length and area of 6-d-old cucumber plants were measured using ImageJ software (version 1.36b; http://rsb.info.nih.gov/ij). The root morphology was analyzed using an Epson Perfection 4990 Photo Scanner (Seiko Epson, Nagano, Japan). Root length, surface area, projArea (sum of the projection roots for all the merged images), and volume were analyzed using WinRHIZO software (2007 version; Saint Foy, Canada). Average number of cells per 0.5 mm^2^ in hypocotyl were counted^16,17^. Root meristem size was assessed as the cell number between the QC and the first elongating cell in the different transgenic cucumber plants^18–20^.

### Microscopy

The hypocotyls of 6-d-old cucumber plants were fixed overnight in a 1:1:18 solution of formaldehyde, acetic acid, and 50% ethanol. The hypocotyls and tip roots were stained for 1 h with 1% safranin in 50% ethanol. After washing in 50% ethanol, the hypocotyls were incubated for 2 h in a solution consisting of 50 mM phosphate buffer (pH 7.2), 4% paraformaldehyde, and 0.25% glutaraldehyde. The samples were washed three times with phosphate-buffered saline (PBS), after which they were embedded in 5% agar containing saffron green. The samples were then sectioned using a vibrating microtome (0.5-cm sections) and visualized using a BX53 microscope (Olympus, Tokyo, Japan).

### Subcellular localization

Strips of onion (*Allium cepa*) bulb epidermis were bombarded with gold particles containing the pUC 35S::*CsGPA1-GFP* plasmid using a PDS-1000/He particle delivery system (Bio-Rad, Hercules, CA, USA) and intraepidermal placed on MS medium and incubated for 24 h at 22 °C in darkness. The strips were then incubated in 3% (w/v) NaCl solution for a few seconds to induce plasmolysis and then analyzed for GFP fluorescence using a C1 confocal laser scanning microscope (Nikon, Tokyo, Japan) set to a 488-nm excitation wavelength.

### Immunolocalization

Cucumber plants were grown until the eight- or nine-leaf stage. Transverse sections were prepared using 1-cm samples collected from the root tip and petiole region. The samples were cut into transverse or longitudinal paraffin sections and placed on microscope slides. After dewaxing, the sections were washed with 0.01 M PBS, pH 7.2–7.4 (137 mm NaCl, 2.7 mm KCl, 10 mm Na_2_HPO_4_, and 2 mm KH_2_PO_4_), containing 0.05% Tween-20. The sections were then incubated in serum blocking solution for 12 h at 4 °C, followed by a 4-h incubation in a solution of antibody that specifically targets CsGPA1 (1:100 dilution). The control sections were treated with pre-immune serum at the same dilution. The samples were washed three times with PBS and then incubated for 2 h in a solution containing a 1:1,000 dilution of alkaline phosphatase-conjugated anti-mouse IgG secondary antibody (PV9002, ZSGB-Bio Company, Beijing, China). The reaction was stopped by washing the sections with distilled water for 5 min. Finally, the stained sections were visualized using a BX53 microscope (Olympus). The N-terminal peptide of CsGPA1 (SRNRHYNEQDAEEKTQAC) was synthesized by Beijing Bormai Biotechnology Co. Ltd. (Beijing, China) and used to produce the antibody with CsGPA1 as the antigen.

### Western blot

The membrane proteins were extracted using a plant membrane protein extraction kit.(Huayueyang Company, Beijing, China,) Briefly, the cucumber samples were ground with liquid nitrogen and then extracted with ice-cold plant lysis buffer with Complete Protease Inhibitor Cocktail Tablets (Roche, Basel, Switzerland). Extracts were incubated on ice for 30 min. Lysed samples were centrifuged at 4 °C at 13,000 g for 20 min to obtain the supernatants as total proteins. The protein level was determined by the BCA method. The same amount of total proteins and extracted membrane proteins were mixed with 5 × loading buffer, then electrophoresed in 10% sodium dodecyl sulfate polyacrylamide gel (SDS-PAGE) and then transferred onto a NC membrane (Millipore, Sigma, Burlington, MA). NC membranes were then blocked with 5% bovine serum albumin (BSA) for 1 h at room temperature. After thrice washing in Tris-buffered saline-Tween 20 (TBST), membranes were incubated with mouse anti-CsGPA1 primary antibody at 1:1000 dilution overnight at 4 °C. After washing in TBST, membranes were incubated with HRP-conjugated secondary antibodies at 1:5000 dilutions for 1 h at room temperature. Finally, proteins were detected using the ECL (Millipore) method. For all samples, protein level of actin was used as the loading control.

## Results

### Identification of CsGPA1

**Figure 1:**
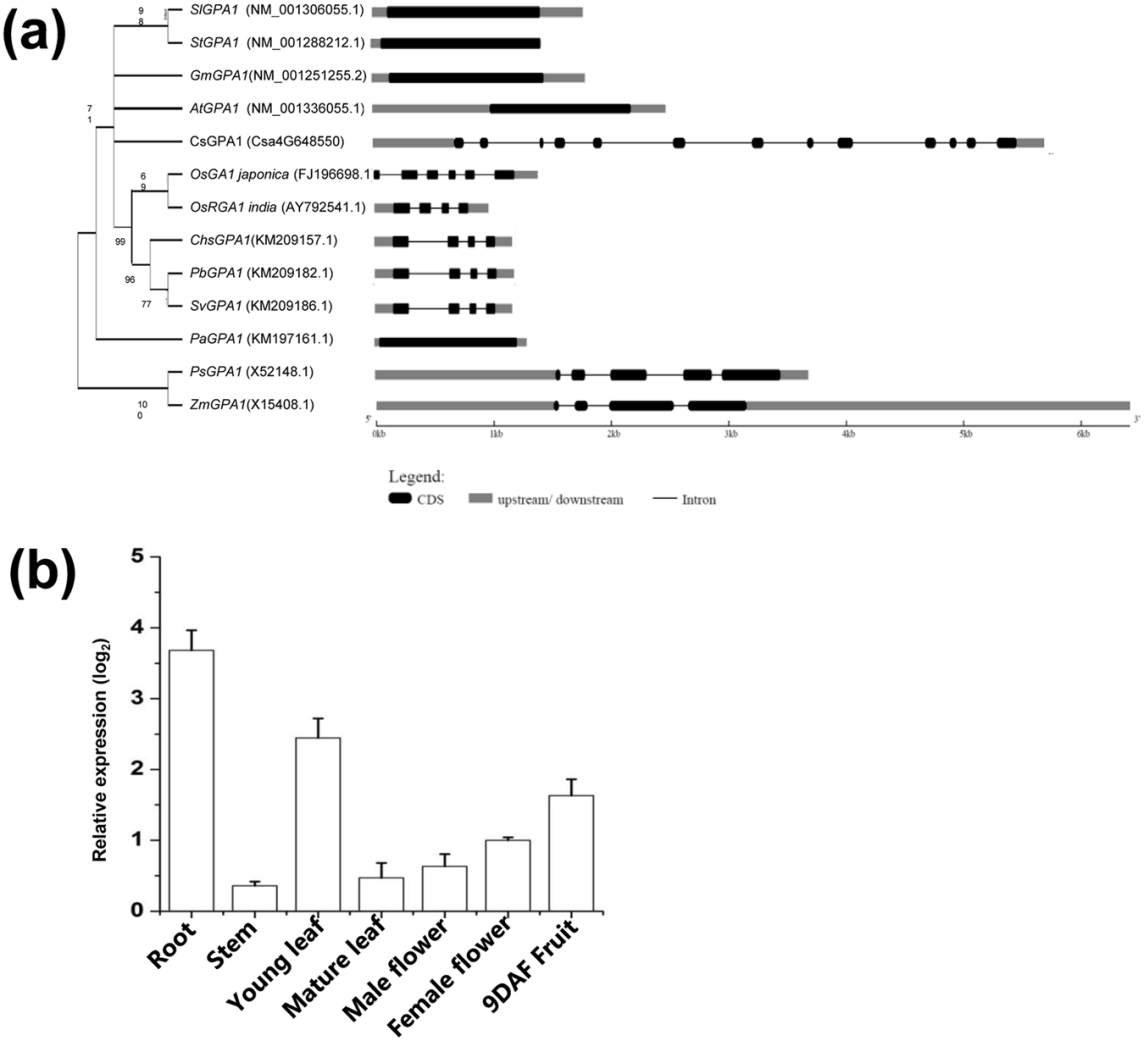
Optimal phylogenetic tree with the sum of branch lengths = 0.2. (**a**) The percentage of replicate trees in which the associated taxa clustered together in the bootstrap test (1,000 replicates) is indicated next to the branches. The evolutionary distances were computed using the Poisson correction method and are indicated as the number of mRNA substitutions per site. Thirteen mRNA sequences were included in the analysis. All positions with gaps and missing data were eliminated. A total of 199 positions were included in the final dataset. Evolutionary analyses were conducted with MEGA7 (Kumar *et al*.^15^). A phylogenetic analysis of the *GPA1* gene was completed using the neighbor-joining method of the MEGA7 software for the following species: *Cucumis sativus* (cucumber), *Zea mays*, *Oryza sativa subsp*. *indica*, *Oryza sativa subsp*. *japonica*, *Pisum sativum*, *Solanum lycopersicum*, *Solanum tuberosum*, *Glycine max*, *Sasa veitchii*, *Chusquea spectabilis*, *Phyllostachys bambusoides* (timber bamboo), *Picea abies* (Norway spruce), and *Arabidopsis thaliana*. The gene structures were generated using an online tool (http://gsds.cbi.pku.edu.cn/). Black boxes and lines represent exons and introns, respectively. Upstream and downstream sequences are indicted by gray boxes. (**b**) Spatiotemporal expression of *CsGPA1* in different cucumber tissues. The data were obtained from three biological replicates, and *CsACTIN* was used as an internal control. DAF, days after flowering.

Using the public BLAST database for cucumber, we obtained a cDNA clone encoding a GPA1-like protein. The 5,879-kb full-length CsGPA1 gene encoded 392 amino acids with a predicted molecular mass of 44.6 kDa. CsGPA1 was located on chromosome 4 and comprised 13 exons and 12 introns, with one putative transmembrane domain (Supplementary Fig. 1a,b). A phylogenetic analysis of full-length amino acid sequences from different species found that the GPA1 genes could be divided into two clades. The first clade consisted of all genes, except for *PsGPA1* and *ZmGPA1*, and showed that *CsGPA1* possessed around 70% evolutionally homology with *Arabidopsis*, *Solanum*, *Glycine max*, *Cucumis sativus* (*cucumber*), *Zea mays*, *Oryza sativa*, and other species (Fig. 1a). Spatiotemporal expression analysis found endogenous *CsGPA1* expression in all examined tissues, and it was particularly high in roots and young leaves (Fig. 1b).

### Subcellular localization and immunohistochemical assay of CsGPA1

**Figure 2:**
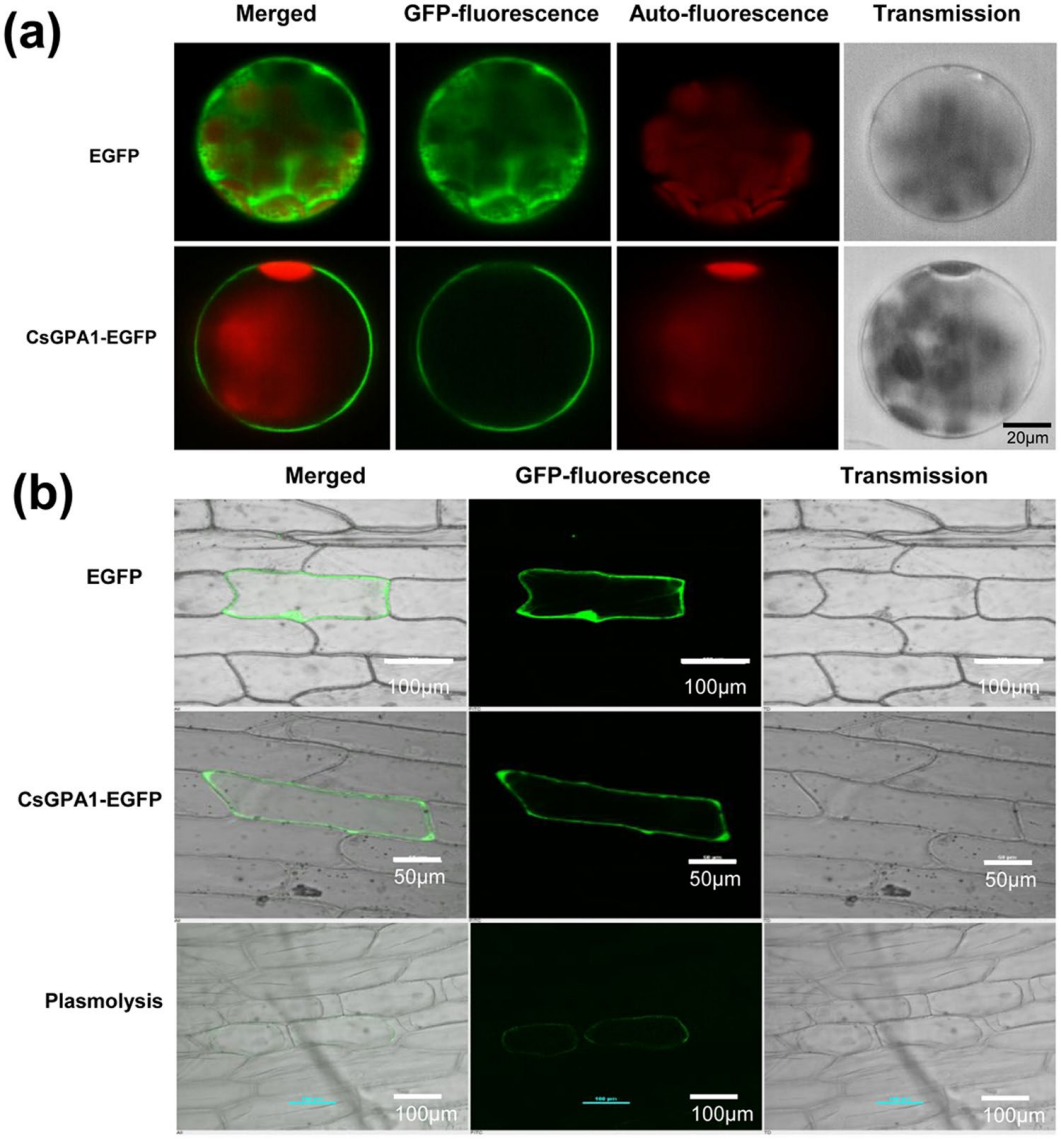
Subcellular localization of CsGPA1-GFP. (**a**) Subcellular localization of CsGPA1-enhanced green fluorescent protein (EGFP) in *Arabidopsis thaliana* mesophyll protoplasts. (**b**) Subcellular localization of CsGPA1-EGFP in onion epidermal cells after plasmolysis. The plasmid encoding EGFP alone served as the control. Green represents EGFP fluorescence, while red represents chlorophyll fluorescence. Scale = 20, 50 and100 µm.

Transient expression assays using *A*. *thaliana* mesophyll protoplasts revealed that CsGPA1-GFP was mainly present in the plasma membrane (Fig. 2a). Similar results were obtained for chloroplast-free *A*. *cepa* epidermal cells (Fig. 2b). The positive control (pUC vector containing unfused GFP) showed fluorescence throughout the *A*. *thaliana* mesophyll protoplasts and *A*. *cepa* epidermal cells.

**Figure 3:**
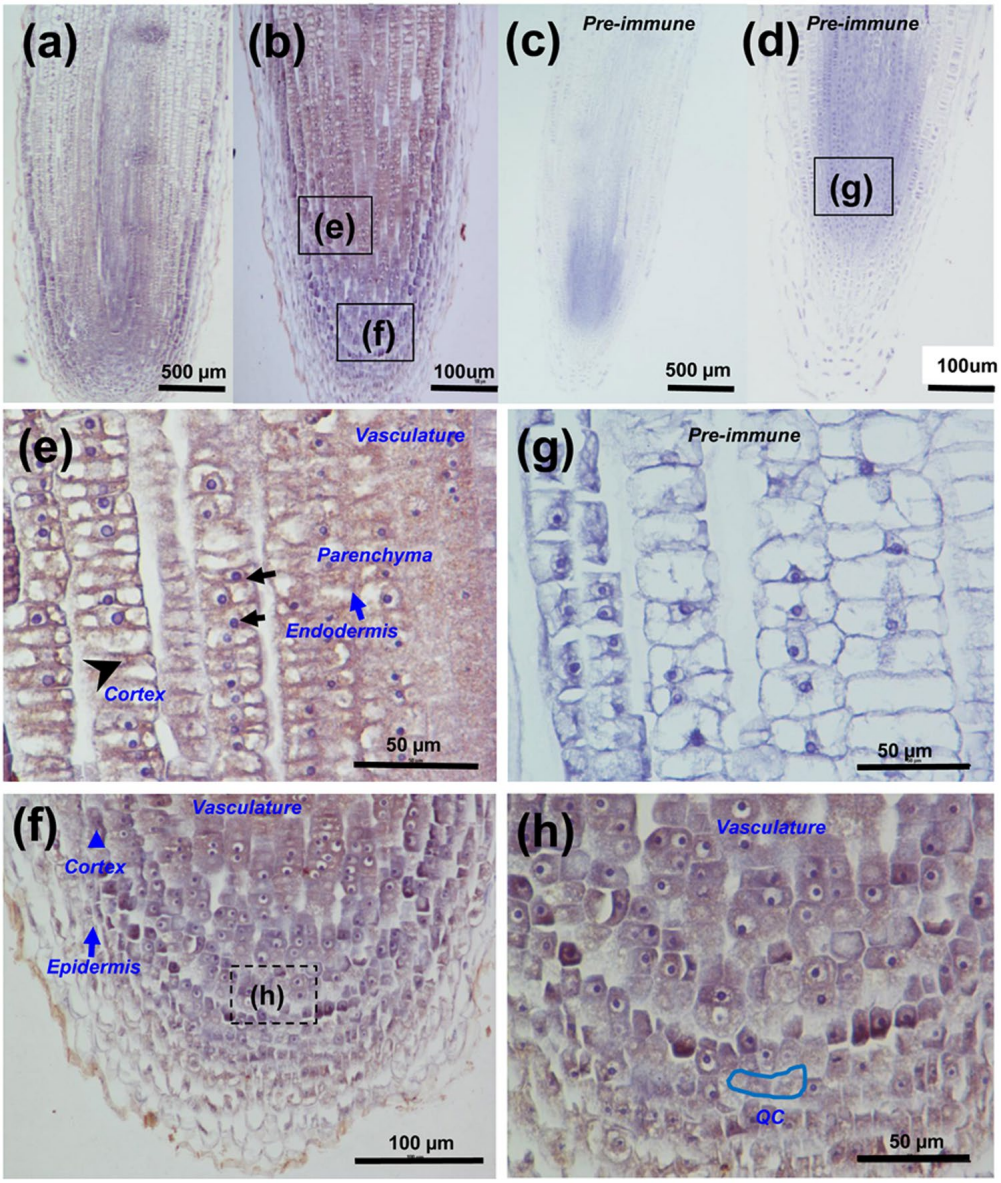
Immunohistochemical analysis of CsGPA1 in cucumber root tips. An anti-CsGPA1 monoclonal antibody (**a**,**b**) and pre-immune serum (**c**,**d**) were used to analyze longitudinal sections of the root tips of 4-leaf-old plants for CsGPA1 expression. Scales = 50, 100 and 500 µm. (**e**–**g**) Magnified views of the images in (**b**,**d**). (**h**) Magnified view of the areas boxed in (**f**), respectively. Black arrows indicate the nucleus, blue arrows indicate the cell layers of the root meristem, and black arrowheads indicate the specific distribution of immunostaining in the root tip cortex as well as epidermal, endodermal, parenchymal and phloem cells. QC, quiescent center.

**Figure 4:**
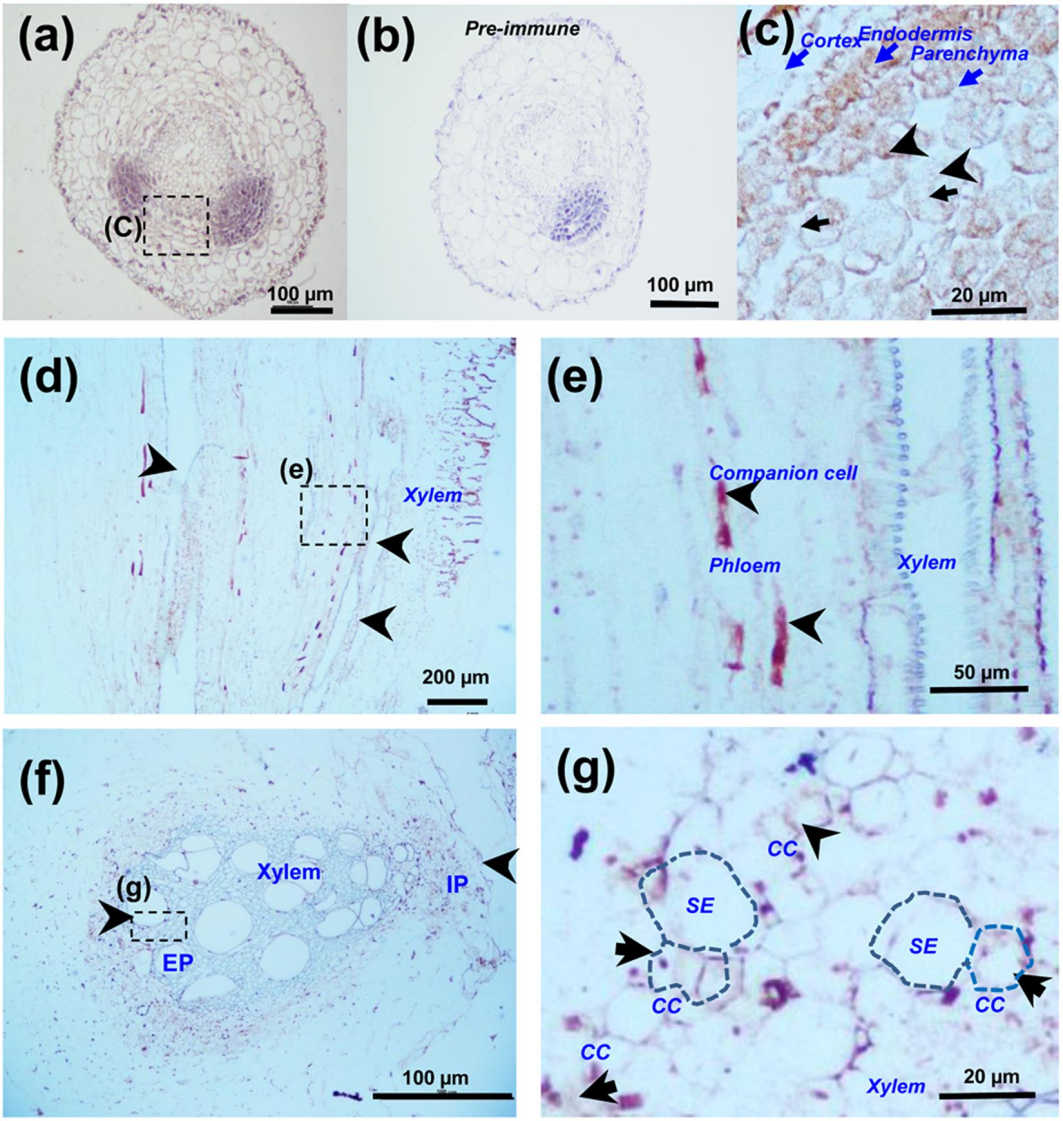
Immunohistochemical analysis of CsGPA1 in cucumber leaves, root tips, and stems. Anti-CsGPA1 monoclonal antibody was used to analyze transverse sections of the root tips of 4-leaf-old plants (**a–c**). Control section treated with pre-immune serum revealing the absence of immunoreactivity in the root tip (**b**). Longitudinal sections of the petioles of 15-d-old plants (**d**,**e**), and stem cross sections of 30-d-old plants (**f**,**g**). (**c**) Magnified view of images in (**a**). (**e**,**g**) Magnified view of the images in the boxes of (d and f). Scale = 20, 50, 100, and 200 µm. Black arrows, nucleus; blue arrows, cell layers of root meristem; black arrowheads indicate the specific distributions of immunostaining in the root tip cortex, epidermal, endodermal, parenchymal, phloem cells, sieve elements, and companion cells. SE, sieve element; CC, companion cells; X, xylem; EP, external phloem; IP, internal phloem.

We also performed an immunohistochemical assay to investigate the subcellular localization of CsGPA1 in 4-leaf-old cucumber plants. Investigation on longitudinal sections of the root tips showed that CsGPA1 was present in the cortical, epidermal, and endodermal vasculature cells of the roots and quiescent center (Fig. 3a,b,f,h). In the magnified images, the immunolabeling signal was detected in the plasma membranes of epidermal, cortical and root cap cells. These findings agreed with the results of the GFP-assay. We also found signals in the cytosol of endodermal, parenchymal, and vasculature cells (Fig. 3a–h), suggesting that CsGPA1 might localize in the cytosol as well. No signal was detected in the nucleus. Investigation on cross sections of the roots also indicated that the CsGPA1 signal was present in the plasma membranes of the cortex and epidermal cells and was present in the cytosol of the endodermis and parenchyma but was excluded from the nucleus (Fig. 4a–c).

In 4-leaf-old cucumber plants, CsGPA1 immunolocalized primarily to the phloem, the companion cells of leaf veins, and to the external and internal phloem of stems (Fig. 4d–f). In particular, CsGPA1 localized to the membrane of sieve elements and the cytosol of companion cells (Fig. 4g). The association of CsGPA1 with the membranes of extension cells (i.e., epidermis, cortex) suggests that it may function in cell expansion and size extension.

### CsGPA1 enhances cucumber seed germination and early seedling growth

**Figure 5:**
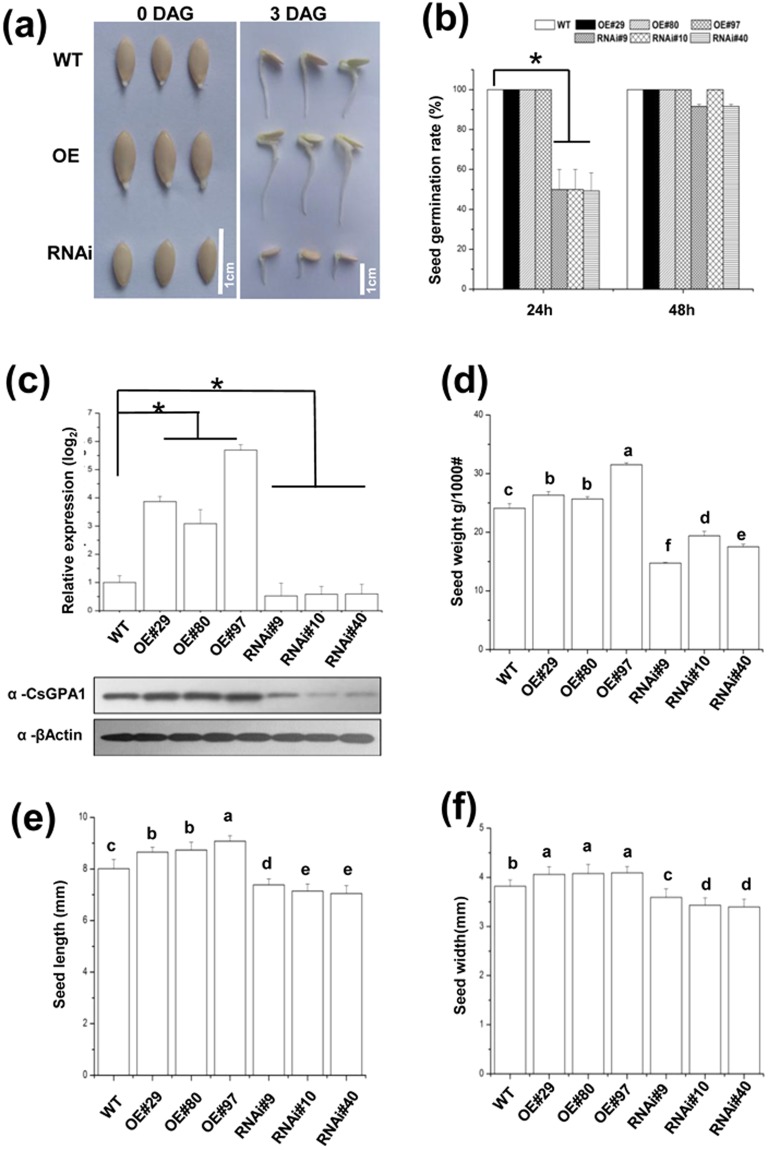
Seed germination in transgenic cucumber lines. (**a**) Images of germinated seeds 0 and 3 d after germination from transgenic cucumber lines. Scale = 1 cm. (**b**) Seed germination rate of transgenic cucumber lines after 24 h and 48 h of darkness. (**c**) Relative *CsGPA1* expression and western blot with anti-CsGPA1 monoclonal antibody at 6 days after germination in different transgenic cucumber lines. (**d**) Weight (g) per thousand seeds of different transgenic cucumber lines. (**e**) Seed length of transgenic cucumber lines. (**f**) Seed width of transgenic cucumber lines. Seeds were considered to have germinated if the emerging radicle grew to 3 mm. Seed germination rates (%) were analyzed at the indicated time points. Data are presented as the mean ± standard deviation of three independent replicates with at least 20 seeds counted per replicate. Values that differed significantly from the WT values are indicated. *p < 0.05 according to a Bonferroni post-hoc test. OE, *CsGPA1-*overexpressing transgenic plants; RNAi, *CsGPA1-*silenced transgenic plants.

To assess whether CsGPA1 contributes to cucumber growth, we constructed several *CsGPA1*-overexpressing (OE) and RNA interference(RNAi)lines (Fig. 5a–f). The qRT-PCR and western blot assays showed that mRNA levels correlated strongly with the levels of CsGPA1 in these six lines (Fig. 5c), and we thus selected them for further study.

We found a difference in germination rates between the OE, RNAi, and Wild-type(WT) lines in darkness (Fig. 5a). Analysis of samples incubated for 24 h under optimal conditions (30 °C in darkness) revealed that 100% of the WT and OE seeds had germinated, but only 49.3% of the RNAi seeds had germinated, although this percentage increased to 91.67% at 48 h (Fig. 4b). These results suggest that RNAi silencing of the CsGPA1 gene delayed speed and reduced rates of seed germination. The CsGPA1 gene and protein expression levels in the OE and RNAi lines were considerably higher and lower, respectively, than that in the WT plants at 6 d after germination (Fig. 5c).

**Figure 6:**
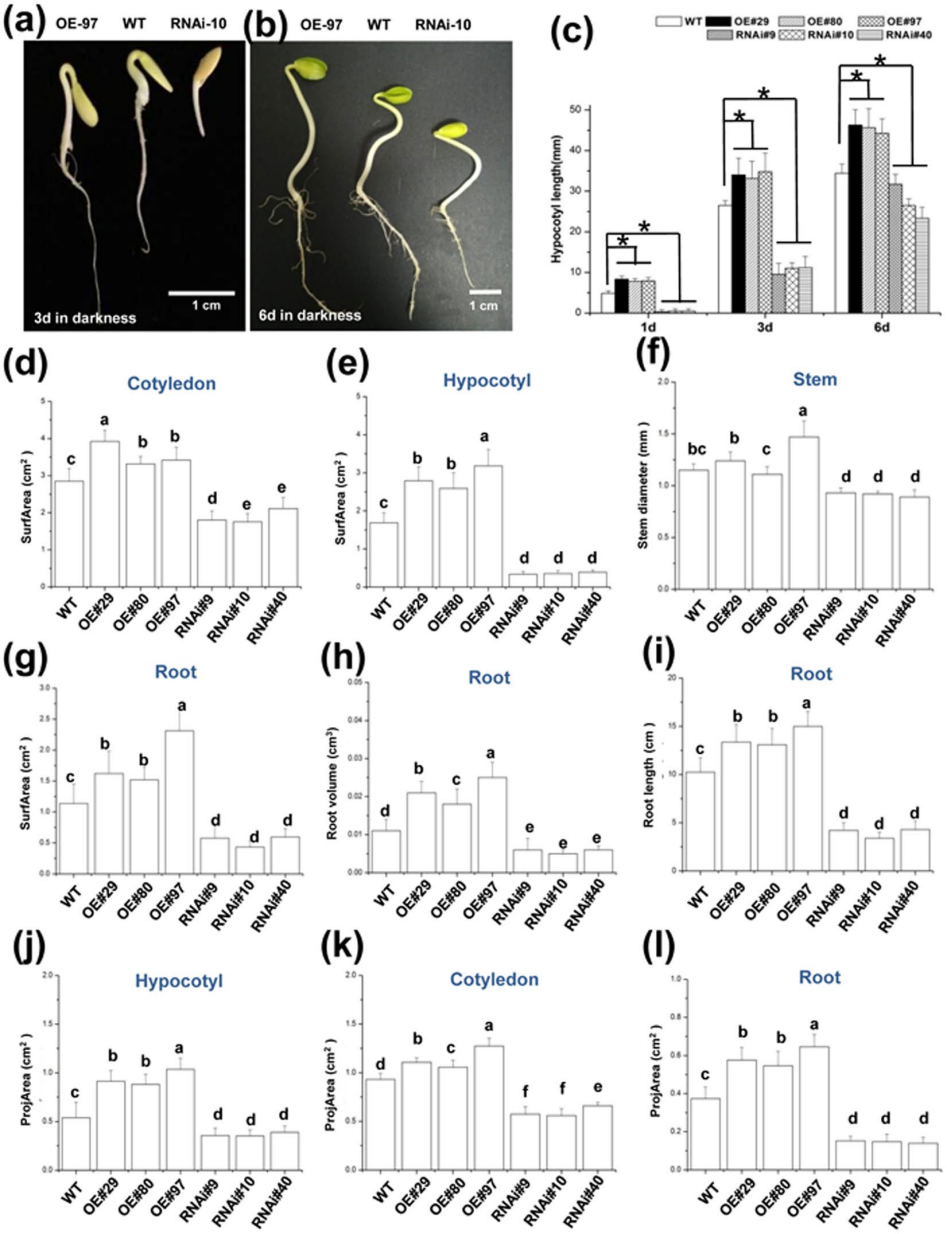
Changes to the morphological characteristics of transgenic cucumber lines. (**a**,**b**) Morphology of 3-d-old (**a**) and 6-d-old (**b**) WT and transgenic seedlings grown in darkness. WT and transgenic seedlings were grown in 9 cm diameter Petri dishes containing two layers of filter paper moistened with 5 mL of sterilized distilled water. Plates were incubated for 6 d at 30 °C in darkness. Seeds were considered to have germinated if the emerging radicle grew to 3 mm. WT (middle), OE (*CsGPA1-*overexpressing transgenic plants, at left), and RNAi (*CsGPA1-*silenced transgenic plants, at right) seedlings. Scale = 1 cm. Hypocotyl (**c**) length and (**e** and **j**) parameters, (**d**,**k**) cotyledon parameters, (**g**–**i** and **l**) root parameters, and (**f**) stem diameter of 6-d-old seedlings. The WT, OE, and RNAi seedlings were grown on plates supplemented with 5 mL of deionized water and then measured. Error bars correspond to the standard error of the mean. Values and data are presented as the mean ± standard deviation of three independent replicates with at least 20 seedlings counted per replicate. Values that differed significantly from the WT values are indicated. *p < 0.05 according to a Bonferroni post-hoc test.

To further clarify the effects of CsGPA1 on seed development and the morphology of young seedlings, we examined seed physiological traits (thousand-seed weight and seed length/weight) and morphological characteristics of 6-d-old seedlings (length, diameter, surface area, projected area, and volumes of the cotyledon, hypocotyl, and roots). We found that overexpression of *CsGPA1* significantly enhanced seed development and resulted in the production of seeds that were larger and heavier than the WT seeds (Fig. 5d–f). After 6 d of incubation in darkness, the OE seedlings had larger cotyledons, longer hypocotyls, and more lateral roots than the WT or RNAi seedlings. Additionally, the lengths, diameters, surfArea, projArea, and volumes of the cotyledons, hypocotyls, and roots were all greater in the OE seedlings than in the WT or RNAi seedlings after 6 d of darkness incubation (Fig. 6a–l). Together, these observations suggest that CsGPA1 may affect cucumber seed development and early seedling growth in darkness by inducing the growth of the cotyledons, hypocotyl, and primary roots.

### CsGPA1 positively regulates hypocotyl and root elongation

**Figure 7:**
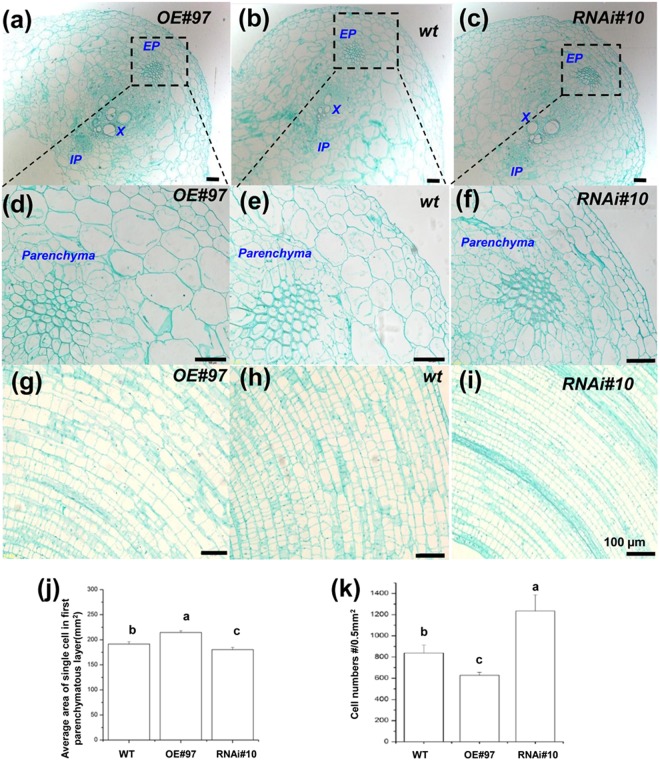
Cross and longitudinal sections of 6-d-old WT and transgenic cucumber seedlings grown in darkness. Hypocotyl cross sections of (**a**) OE line 97, (**b**) WT, and (**c**) RNA line 10. Magnified images of the hypocotyl cross sections of (**d**) OE line 97, (**e**) WT, and (**f**) RNAi line 10 were analyzed. Scale = 100 µm. EP, external phloem; IP, internal phloem; X, xylem; PP, parenchymal cell; BS, vascular bundle sheath; CC, companion cell; SE, sieve element. Longitudinal sections of the hypocotyls in (**g**) OE line 97, (**h**) WT, and (**i**) RNAi line 10. The dotted lines indicate the direction of the bending and the arrows indicate sieve element cells. (**j**) Average single cell area in the first layer of parenchymal cells in hypocotyl. (**k**) Average number of cells per 0.5 mm^2^ in hypocotyl. (n = three visual fields). Values that differed significantly from the WT values are indicated. *p < 0.05 and according to a Bonferroni post-hoc test.

Plant growth involves both cell elongation and proliferation. We assessed the function of CsGPA1 in cell elongation and/or proliferation in the hypocotyls and roots of OE, WT, and RNAi seedlings (Fig. 6a–l). We examined transverse and longitudinal tissue sections of seedlings and hypocotyls. Epidermal, parenchymal, vascular bundle, and external phloem cell sizes were compared between the different lines using microscopic images. Analyses of the hypocotyl cross sections revealed that the external phloem cells were significantly larger in the OE seedlings than in the WT seedlings. Furthermore, the hypocotyls of OE plants had more epidermal and xylem cells than those of WT plants and had more loose cell layers (epidermis, parenchyma, vascular bundle, and external phloem) that were more disorganized (Fig. 7a,d,g). In contrast, the external phloem cells of the RNAi lines were smaller than those of the WT seedlings. In addition, the RNAi plants had fewer epidermal and xylem cells (Fig. 7b,e,and h) and had more tightly packed cell layers than did the WT hypocotyls (epidermis, parenchyma, vascular bundle, and external phloem; Fig. 7c,f,i–k).

An examination of the cotyledon and root longitudinal sections provided additional evidence of differences between the lines (Supplementary Fig. 2a–j). The cells of the OE seedlings expanded relatively quickly, resulting in larger but fewer cells in the meristem zone of the roots in comparison with the WT and RNAi lines (from QC to the first elongation cell) (Supplementary Fig. 3a–d). In contrast, the RNAi root and cotyledon cells divided and expanded relatively slowly. Analyses of the microscopic images suggested that the enlarged cotyledons, longer hypocotyls, and greater number of lateral roots in the OE lines might be the result of increased cell elongation. In contrast, the smaller cotyledons, shorter hypocotyls, and fewer lateral roots observed in the RNAi lines might be a consequence of decreased elongation. Thus, CsGPA1 may function as a positive regulator of seedling cell elongation in darkness, which then accelerates subsequent plant growth.

## Discussion

Our study describes an entirely novel G protein from cucumber, a global food crop, and helps to elucidate the roles of G proteins in plants, particularly those both internal and external phloem. G-protein signaling pathways are known to affect many plant growth and development processes. Mutant analyses have shown that heterotrimeric G-proteins represent a critical nexus in the signal regulation of a variety of processes such as germination, cell division and elongation, stress responses, and plant morphology^1^. In this study, we found that the *CsGPA1* gene was homologous to the *GPA1* gene in other plant species. GPA1 is expressed in all stages of development and in all organs, with the exception of mature seeds^21^. Among the measured samples, expression was highest in the root, followed by the stem apex, hypocotyls, cotyledon, and leaf^22^, and levels in immature organs were higher than in mature organs^21^. Similarly, our study demonstrated the spatiotemporal expression of endogenous *CsGPA1* (Fig. 1b). Previous studies showed that the Gα subunit was present in the plasma membranes of the pollen tube in other species^23,24^. Our study demonstrated that CsGPA1 is also present in the plasma membrane of the epidermis and cortex in cucumber (Fig. 3a–h), where it may help to regulate early seedling development. Immunohistochemical analyses showed that functional CsGPA1 acting in the epidermal and cortex membranes may affect seedling root growth. CsGPA1 was in the cytosol of the endodermal, parenchymal, vascular, and root meristematic cells (Fig. 4a–g). Gα-null mutations decrease root growth in *Arabidopsis*, rice, and maize^25–27^ and can lead to an increase in cell division to promote primary root elongation^26^ or to a decrease in cell division at the root apical meristem^27^. The results of our seed germination and seedling morphogenesis analyses in OE and RNAi plants showed that *CsGPA1* affects seed germination and early seedling growth in the cotyledons, hypocotyl, and roots (Figs 5a–f and 6a–l). RNAi silencing of the CsGPA1 gene delayed the speed and reduced rates of seed germination (Fig. 5b), inhibited the size of the cotyledons, the lengths of the hypocotyl and the primary root, and the growth of lateral roots (Fig. 6c–l). Thus, we conclude that CsGPA1 is involved in regulation of seed germination and early seedling development in cucumber.

Cell division and elongation are two fundamental cellular processes in the life cycle of plants^9^ regulated in part by the Gα subunit. Mutations to Gα result in inhibition of cell division along the leaf length but not along the leaf width^28,29^ and can lead to dwarfism and etiolated seedling phenotypes in several species^9,11,15^. Maize Gα regulates and enlarges the shoot apical meristem (SAM)^28^, but *gpa1* mutants display no obvious change in SAM height^30^. These findings suggest that the Gα subunit specifically mediates cell proliferation in the shoot apical meristem and leaf primordia but not at the leaf plate meristem. Our study showed that CsGPA1 helps facilitate the elongation of epidermal and parenchymal cells by positively regulating the hypocotyl and root meristem zone in early cucumber seedlings (Fig. 7a–g, Supplementary Figs 2a–i, and 3a–d). This may be conducive to the transport of nutrients from the roots to the upper leaves, as well as the transport of nutrients from the upper part of the cotyledon to the lower part of the roots, and may explain why the leaf areas and root volumes were greater in the OE seedlings than in the WT seedlings. In contrast, the relatively low abundance of CsGPA1 in the RNAi seedlings inhibited the development of the hypocotyl tissue, in particular parenchymal cells surrounding the vascular bundles. This inhibition likely hindered the transport of nutrients through the hypocotyl and suppressed leaf and root growth.

In conclusion, we infer that CsGPA1 is involved in the regulation of meristem cell elongation in the early seedling stage by regulating hypocotyl elongation and root growth. Strong development of early seedlings will be useful for alleviating abiotic stress and enhancing stress tolerance in the cucumber production process in greenhouses during the winter^31^, and thus additional work is required in this direction. Further study of CsGPA1 will be required to elucidate its precise function and will provide insights into the relationship between signal transduction and growth and the development of cucumber. This additional work will serve to increase global food security by creating a more reliable growth system for cucumber and other crops^32–37^.

## Electronic supplementary material

Dataset 1
