# Supplementary material for: Functions of CsGPA1 on the hypocotyl elongation and root growth of cucumbers
Source: Sci Rep. 2018 Oct 22;8:15583. doi: 10.1038/s41598-018-33782-4 (PMC6197229; doi:10.1038/s41598-018-33782-4)
Supplement: Supplementary file 1 — Dataset 1 [file 41598_2018_33782_MOESM1_ESM.pdf]

Functions of CsGPA1 on the hypocotyl elongation and root growth of cucumbers

Authors: Yan Yan[1,2]†, Wenna Zhang[1]†, Yansu Li[2], Chaoxing He[2], Lihong Gao[1]*, Xianchang Yu[2]*

Supplemental Figure 1

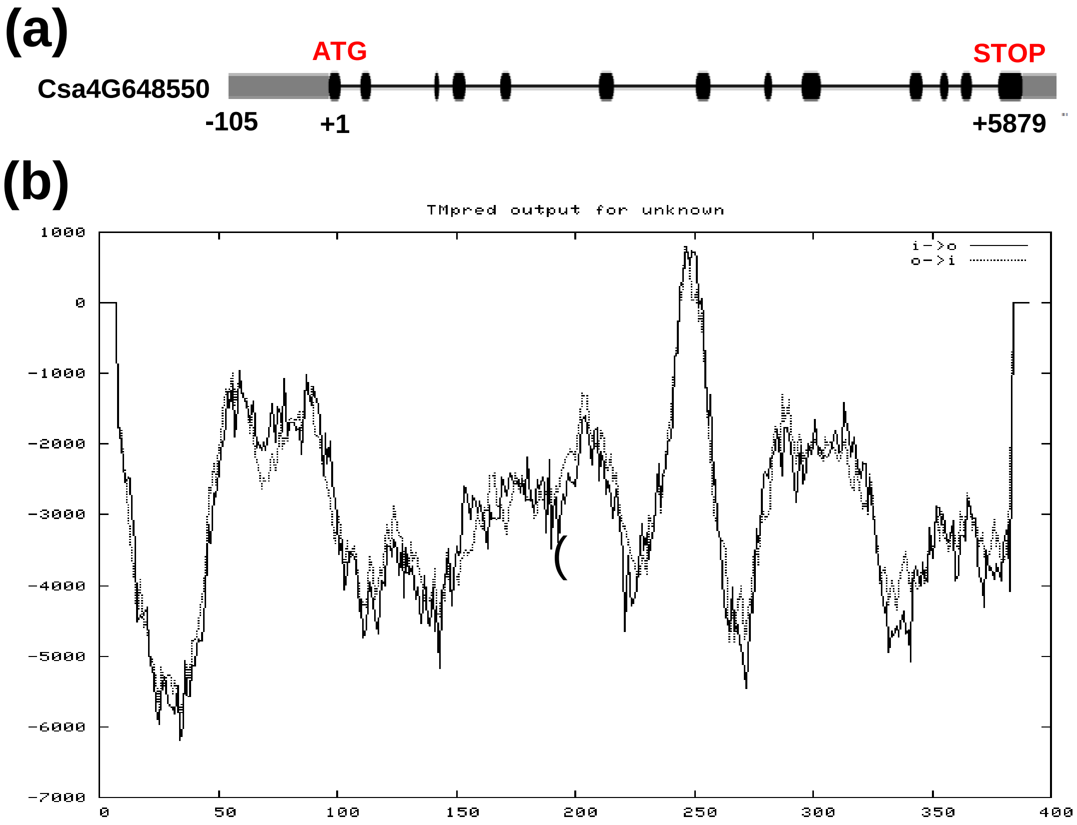

Supplementary Figure 1. GPA1 sequence analysis and identification of potential transmembrane domains in the encoded protein. (a) Exons and introns are represented by black boxes and connecting lines, respectively. The number of exon nucleotides are indicated above the boxes. Numbers in parentheses indicate the length of coding sequences. (b). Hydrophobicity plot analysis of GPA1 using the TMPRED tool (http://www.cbs.dtu.dk/services/TMHMM/). Putative transmembrane domains were detected by searching for regions with a hydrophobicity index greater than 500.

Supplemental Figure 2

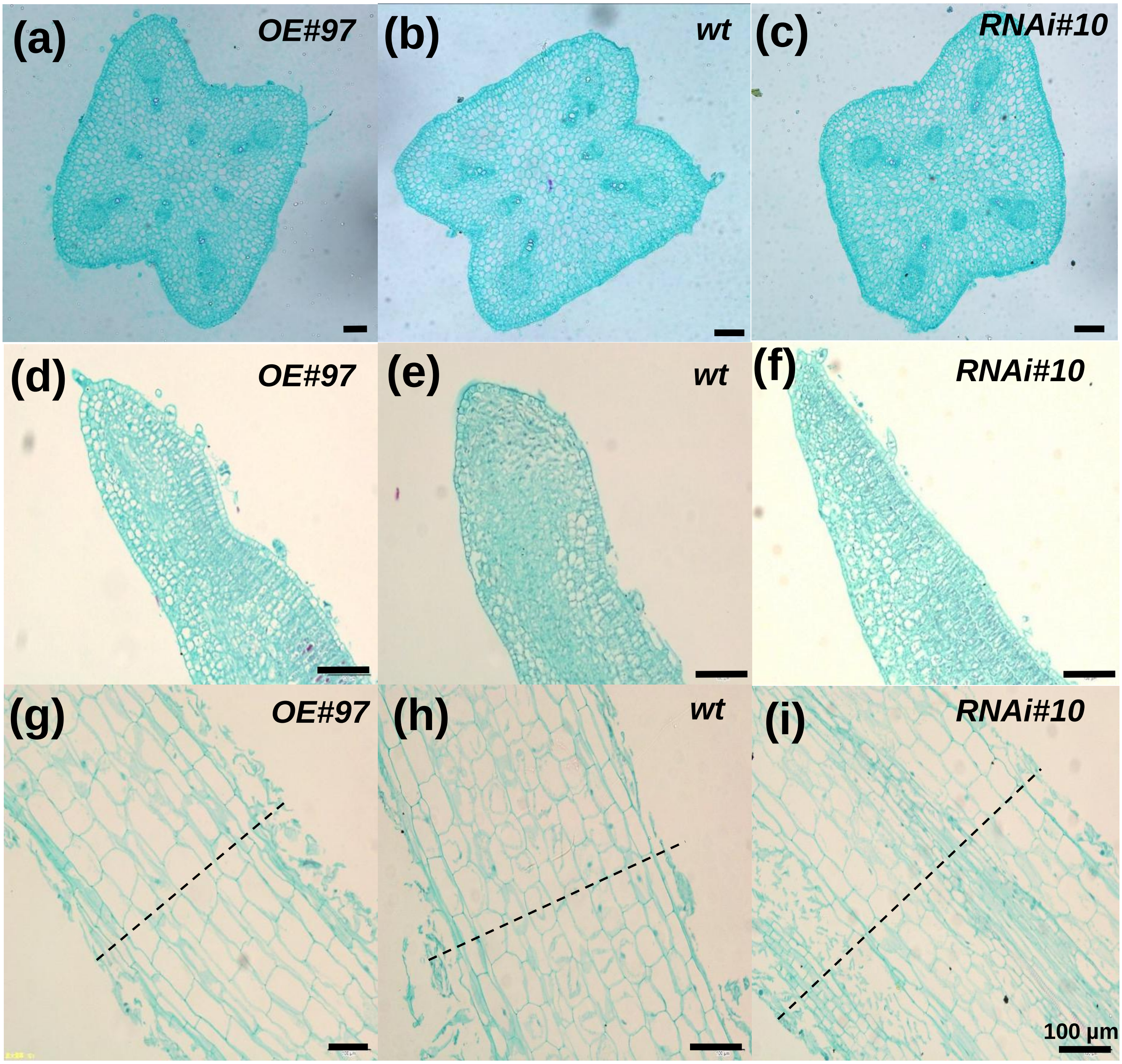

Supplementary Figure 2. Cross and longitudinal sections of 6-d-old WT and transgenic cucumber seedlings grown in darkness. Hypocotyl cross sections of (a) OE, (b) WT, and (c) RNAi plants. Cotyledon longitudinal sections of (d) OE, (e) WT, and (f) RNAi plants. Root tip longitudinal sections of (g) OE, (h) WT, (i) RNAi plants. Scale = 100 µm.

Supplemental Figure 3

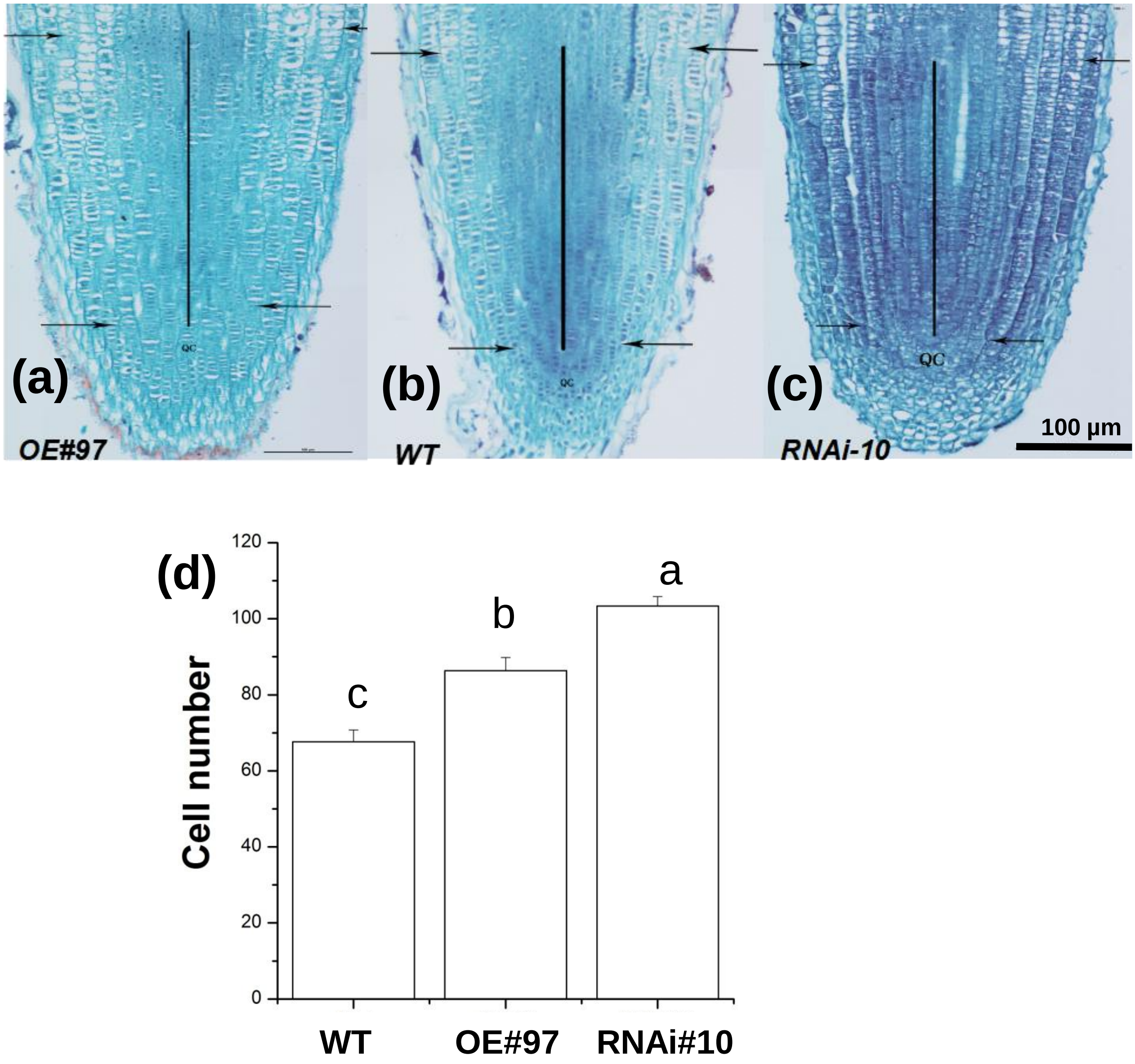

Supplementary Figure 3. longitudinal sections of 6-d-old WT and transgenic cucumber seedlings grown in darkness. Region from QC to the first elongated cell in OE line 97, WT and RNAi line 10;Root tip longitudinal sections of (a) OE, (b) WT, (c) RNAi plants. Scale = 100 µm.(d)Average number of cells numbers in the root meristem zone (From QC to the first elongated cell) . (n = three different seedlings)

Supplemental Figure 4

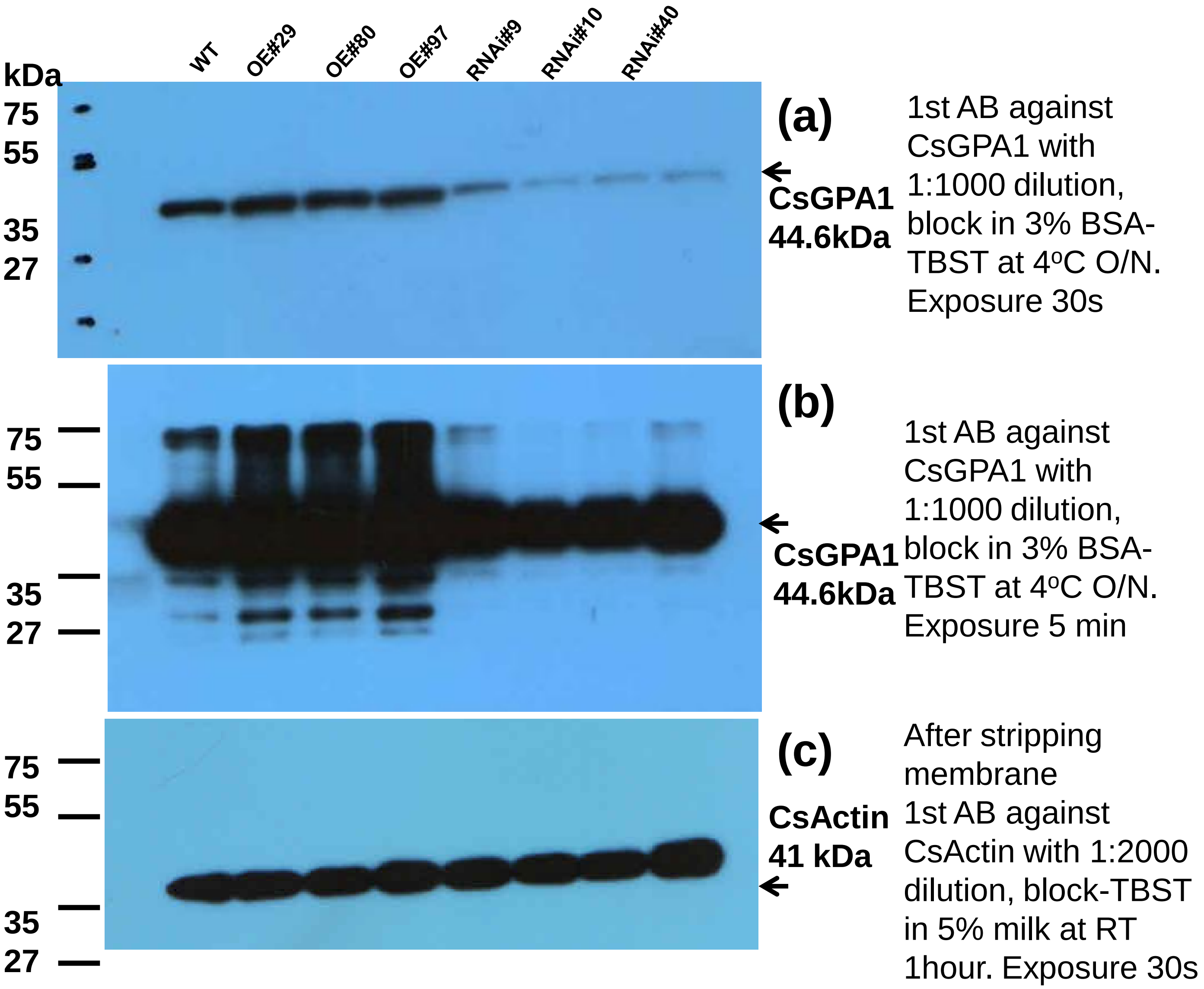

Supplementary Figure 4. Expression of CsGPA1 by Western blot in 6-day-old different transgenic cucumber lines.
The WT, overexpression and RNAi samples of transgenic cucumber lines were collected for membrane proteins extraction. The expression of CsGPA1 were detected by W-B. The band of each samples were visualized by ECL for exposure of 30s (a) and 5 min (b) on film. The protein level of CsActin was used as a loading control for exposure of 30s by ECL (c) after membrane stripping.

Supplemental Table 1

Supplemental Table 1. Oligonucleotides list used in the study

**Gene**	**Name and sequence**	**Size of RT-PCR fragment**
CsGPA1 clone forward	F 5′-ATGCTGTCTCATTTGAGTAGAAA-3′	1179 bp
CsGPA1 clone reverse	R 5′-TCACAATAACCCAGCCTCA-3′	
CsGPA1 clone with *SmaI*	F 5′-TCCCCCGGGATGCTGTCTCATTTGAGTAGAAA-3′	1196 bp
CsGPA1 clone with *XbaI*	R 5′-GCTCTAGATCACAATAACCCAGCCTCA-3′	
CsGPA1 RNAi upstream clone with *AscI*	5′-AGGCGCGCCAGTAGATCGGGTGTTTAAGGTATAC-3′	129 bp
CsGPA1 RNAi upstream clone with *SwaI*	5′-GATTTAAATTCACAATAACCCAGCCTCAAA-3′	
CsGPA1 RNAi downstream clone with *SpeI*	5′-GACTAGTAGTAGATCGGGTGTTTAAGGTATAC-3′	129 bp
CsGPA1 RNAi downstream clone with *BamHI*	5′-CGGGATCCTCACAATAACCCAGCCTCAAA-3′	
CsGPA1 subcellular localization clone with *XbaI* forward	5′-GCTCTAGAATGCTGTCTCATTTGAGTAGAAA-3′	1196 bp
CsGPA1 subcellular localization clone with *BamHI* reverse	5′-CGGGATCCCAATAACCCAGCCTCA-3′	
CsGPA1 qRT-PCR	F 5’- ACCGAATGATGGAGACGAAGGAA-3’	144bp
	R 5'- ACCATTCACACACACTGAGAGGGA -3'	
CsACTIN qRT-PCR	F 5’- TTCTGGTGATGGTGTGAGTC-3’	260 bp
	R 5'- GGCAGTGGTGGTGAACATG-3'	
